# Clinical Characteristics and Prognostic Factors in Patients With Sepsis: A Retrospective Study

**DOI:** 10.1111/jcmm.71005

**Published:** 2026-01-26

**Authors:** Mengxia Yang, Tengfei Chen, Junhao Liu, Xiaolin Wang, Xuerui Wang, Xiaolong Xu, Qingquan Liu

**Affiliations:** ^1^ Beijing Hospital of Traditional Chinese Medicine Capital Medical University Beijing China; ^2^ Beijing Institute of Traditional Chinese Medicine Beijing China; ^3^ Graduate School of Beijing University of Chinese Medicine Beijing China; ^4^ Affiliated Hospital of Changzhi Research Institute of Traditional Chinese Medicine Changzhi China; ^5^ Clinical Diagnosis, Treatment and Research Center of Sepsis of Traditional Chinese Medicine Capital Medical University Beijing China; ^6^ Beijing Key Laboratory of Innovative Research on Removing Stasis and Detoxification Theory in Infectious Diseases Beijing China

**Keywords:** 28‐day prognosis, predictive efficacy, retrospective analysis, risk factors, sepsis

## Abstract

This retrospective study aimed to investigate the risk factors influencing the 28‐day clinical prognosis of sepsis patients and evaluate their predictive efficacy. Clinical data of patients diagnosed with sepsis between January 1, 2019, and December 31, 2023, were collected from the Hospital Information System (HIS) of Beijing Hospital of Traditional Chinese Medicine, Capital Medical University. Based on 28‐day outcomes, patients were divided into survival (*n* = 146) and death (*n* = 81) groups. Statistical analysis was performed using SPSS 20, employing univariate and multivariate logistic regression to identify prognostic risk factors. Receiver operating characteristic (ROC) curve analysis was conducted to assess the predictive performance of these factors, with the area under the curve (AUC) calculated for evaluation. Although blood stasis syndrome was not included in the final model due to collinearity with critical indicators, univariate analysis demonstrated its significant prognostic value (OR = 2.49, 95% CI 1.199–5.17, *p* = 0.014), and ROC curve analysis confirmed its fundamental discriminatory capacity (AUC > 0.5). Multivariable logistic regression identified CRP, TT, disease severity, CA and ARDS as independent risk factors for sepsis mortality. ROC analysis showed all individual indicators and the combined model had AUC > 0.5, with the combined model achieving the highest AUC. The combined model demonstrated good stability via Hosmer‐Lemeshow testing (*p* = 0.067). This study established CRP, TT, disease severity, CA and ARDS as independent mortality risk factors in sepsis, with the combined model showing optimal performance. It demonstrated consistency between TCM macro‐pattern differentiation and Western medical indicators, providing a framework for integrated prognostic models that combines both medical approaches.

## 1 Introduction

According to the 2016 international consensus jointly issued by the European Society of Intensive Care Medicine and the Society of Critical Care Medicine, sepsis is defined as a life‐threatening organ dysfunction caused by a dysregulated host response to infection, characterised by high morbidity and mortality [1, 2]. Updated epidemiological data from the 2020 Global Burden of Disease study revealed that approximately 49 million people worldwide suffer from sepsis annually, with about 11 million deaths attributed to sepsis and its complications, accounting for 19.7% of global all‐cause mortality [3]. Over the past decade, with the progressive clinical implementation of treatment protocols recommended in the *Surviving Sepsis Campaign Guidelines*, multiple medical institutions have reported a decline in sepsis‐related mortality from an initial rate of 37% to the current 30% [4]. Nevertheless, this mortality rate remains unacceptably high [5]. Additionally, the incidence of sepsis among hospitalised patients has shown a near‐doubling trend during the same period [6]. Studies suggest that the high mortality of sepsis is attributable to its atypical clinical manifestations, underscoring the critical importance of early assessment, diagnosis and treatment in improving patient outcomes [7, 8].

Although there is no explicit record of the term ‘sepsis’ in the classical texts of Traditional Chinese Medicine (TCM), based on its clinical manifestations and disease progression, it is generally categorised under the TCM syndromes of ‘internal collapse of gangrenous toxin (ju du nei xian)’, ‘bi syndrome (bi zheng)’, ‘warm disease (wen bing)’ and ‘yellowing of furuncle (ding chuang zou huang)’. The aetiology and pathogenesis primarily revolve around four factors: ‘toxin’, ‘heat’, ‘stasis’ and ‘deficiency’ [9, 10]. Regarding the pattern differentiation and treatment of sepsis, a relatively mainstream viewpoint is the ‘four syndromes and four methods’ proposed by Professor Wang Jinda. The four syndromes include: toxin‐heat syndrome, blood stasis syndrome, acute deficiency syndrome and bowel qi obstruction syndrome. The corresponding four therapeutic methods are heat‐clearing and toxin‐resolving (qing re jie du) method, blood‐activating and stasis‐dispelling (huo xue hua yu) method, strengthening the body resistance and consolidating the root (fu zheng gu ben) method and purgation and downward‐draining method (tong li gong xia fa) [11].

Based on this framework, this study aims to collect medical records of sepsis inpatients from the Hospital Information System (HIS) of Beijing Hospital of Traditional Chinese Medicine, Capital Medical University, between January 1, 2019, and December 31, 2023. The study will further investigate the impact of factors such as infection site, disease severity, TCM syndrome types and laboratory indicators on clinical outcomes, with the goal of providing valuable insights for future sepsis diagnosis and treatment.

## 2 Research Centre

### 2.1 Study Design

A retrospective case–control study.

#### 2.1.1 Data Source

Patient data were extracted from the HIS of Beijing Hospital of Traditional Chinese Medicine, Capital Medical University, including hospitalised patients admitted between January 1, 2019, and December 31, 2023. This study was approved by the Ethics Committee of Beijing Hospital of Traditional Chinese Medicine, Capital Medical University (Approval No. 2024BL02‐099‐01).

## 3 Study Protocol

### 3.1 Case Selection Criteria

#### 3.1.1 Western Medicine Diagnostic Criteria

Patients must meet the diagnostic criteria established in The Third International Consensus Definitions for Sepsis and Septic Shock (Sepsis‐3), published on February 23, 2016: confirmed infection + SOFA score ≥ 2 points [1].

#### 3.1.2 TCM Diagnostic Criteria

Diagnosis should comply with the criteria outlined in *Integrated Traditional Chinese and Western Medicine Therapy for Sepsis* (2008 National Conference on Critical Care and Emergency Medicine). The TCM syndrome differentiation includes the following:

Toxin‐Heat Syndrome—high fever, thirst with desire to drink, abdominal distension with constipation, red tongue with yellow coating, surging and rapid or thin and rapid pulse and abnormal peripheral white blood cell count; acute deficiency syndrome—pale complexion, cold and clammy extremities, profuse sweating, oliguria, thin and rapid or fading pulse and hypotension; blood stasis syndrome—fixed tenderness, haemorrhage, cyanosis, dark purple tongue and abnormalities in hemorheology, coagulation or fibrinolysis systems; bowel qi obstruction syndrome—abdominal distension, vomiting, absence of bowel movements or flatus and weakened or absent bowel sounds [12].

#### 3.1.3 Inclusion Criteria

Meets both Western and TCM diagnostic criteria above; age ≥ 18 years; complete medical records available.

#### 3.1.4 Exclusion Criteria

Does not meet inclusion criteria; incomplete medical records; pregnant or lactating women.

### 3.2 Grouping Method

Patients were stratified into survival and death groups based on their 28‐day post‐admission outcomes.

### 3.3 Data Collection

#### 3.3.1 Outcome Indicators

28‐day clinical outcomes (survival status).

#### 3.3.2 General Conditions

Included gender, age and vital signs: temperature (Temp), heart rate (HR), respiratory rate (RR) and mean arterial pressure (MAP).

#### 3.3.3 Comorbidities

Old myocardial infarction (OMI), acute myocardial infarction (AMI), heart failure (HF), cardiac arrest (CA), hypertension, atrial fibrillation (AF), acute respiratory distress syndrome (ARDS), pneumonia, chronic obstructive pulmonary disease (COPD), cerebrovascular disease (CVD), chronic renal failure (CRF), acute kidney injury (AKI), diabetes mellitus (DM), hypothyroidism and liver injury.

#### 3.3.4 Infection Sites

Respiratory tract infection (RTI), urinary tract infection (UTI), bloodstream infection (BLI), gastrointestinal tract infection (GI) and skin and soft tissue infection (SSTI).

#### 3.3.5 Length of Hospital Stay

Patients's length of stay (LOS) in the ICU.

#### 3.3.6 Disease Severity

Patients were classified as having either sepsis or septic shock based on the presence of shock.

#### 3.3.7 TCM Syndrome Differentiation

According to TCM diagnostic criteria, patients were categorised into four syndrome patterns: toxin‐heat syndrome, blood stasis syndrome, intestinal obstruction syndrome and acute deficiency syndrome.

#### 3.3.8 Laboratory Parameters

Inflammatory markers: C‐reactive protein (CRP) and procalcitonin (PCT). Blood routine: white blood cell count (WBC), neutrophils (NEUT), lymphocytes (LYMPH), red blood cell count (RBC), haemoglobin (HGB) and platelet count (PLT). Coagulation index: prothrombin time (PT), activated partial thromboplastin time (APTT), thrombin time (TT), fibrinogen (FIB), fibrinogen degradation products (FDP) and D‐dimer (D‐D).

### 3.4 Statistical Analysis

Statistical analyses were performed using SPSS software (version 20.0). For continuous variables: normally distributed data were analysed using the independent samples *t*‐test and presented as mean ± standard deviation (x̄ ± s); non‐normally distributed data were analysed using the Mann–Whitney *U* test and expressed as median (interquartile range) [M (P25, P75)]. Categorical variables were compared between groups using the chi‐squared test or Fisher's exact test, as appropriate, with results reported as frequencies (percentages) [*n* (%)]. Univariate and multivariate logistic regression analyses were employed to identify risk factors influencing clinical outcomes in sepsis patients. The predictive performance of significant risk factors was evaluated by constructing receiver operating characteristic (ROC) curves and calculating the corresponding area under the curve (AUC). A *p*‐value < 0.05 was considered statistically significant.

## 4 Results

### 4.1 Comparison of Baseline Characteristics

This study ultimately included 227 sepsis patients (135 males and 92 females). The results demonstrated statistically significant differences (all *p* < 0.05) between the survival and death groups in the following variables: age, MAP, CA, ARDS, pneumonia, AKI, liver injury, RTI, BLI, SSTI, disease severity (sepsis vs. septic shock), blood stasis syndrome, acute deficiency syndrome, PCT, WBC, NEUT, PT, APTT, TT, FIB, FDP and D‐D. In contrast, no statistically significant differences (all *p* > 0.05) were observed between the two groups in terms of gender, body temperature, HR, RR, OMI, AMI, HF, hypertension, AF, COPD, CVD, CRF, DM, hypothyroidism, UTI, GI infection, ICU LOS, toxin‐heat syndrome, intestinal obstruction syndrome, CRP, LYMPH, RBC, HGB or PLT. Detailed results are presented in Table 1.

**TABLE 1:** Baseline characteristics of the study population.

General data	Survival (*n* = 146)	Death (*n* = 81)	Overall (*n* = 227)	*χ* ^2^/*Z*	*p*
Gender					
Male	83 (56.8)	52 (64.2)	135 (59.5)	1.167	0.28
Female	63 (43.2)	29 (35.8)	92 (40.5)		
Age (years)	78 (67, 87)	84 (75, 89)	81 (70, 88)	−3.065	0.002
Vital signs					
Temp (°C)	36.7 (36.3, 37.4)	36.6 (36.3, 37.5)	36.6 (36.3, 37.4)	−0.271	0.787
HR	85 (77, 100)	94 (78, 110)	88 (78, 103)	−1.511	0.131
RR	20 (18, 24)	21 (18, 27)	20 (18, 25)	−1.594	0.111
MAP (mmHg)	93 (84, 101)	85 (74, 96)	91 (80, 100)	−3.264	0.001
Comorbidity					
OMI (%)	12 (8.2)	6 (7.4)	18 (7.9)	0.047	0.828
AMI (%)	12 (8.2)	10 (12.3)	22 (9.7)	1.014	0.314
HF (%)	70 (47.9)	47 (58.0)	117 (51.5)	2.119	0.145
CA (%)	14 (9.6)	26 (32.1)	40 (17.6)	18.185	< 0.001
Hypertension (%)	95 (65.1)	55 (67.9)	150 (66.1)	0.666	0.187
AF (%)	45 (30.8)	30 (37.0)	75 (33.0)	0.91	0.34
ARDS (%)	14 (9.6)	22 (27.2)	36 (15.9)	12.054	0.001
Pneumonia (%)	105 (71.9)	75 (92.6)	180 (79.3)	13.564	< 0.001
COPD (%)	11 (7.5)	10 (12.3)	21 (9.3)	1.437	0.231
CVD (%)	70 (47.9)	38 (46.9)	108 (47.6)	0.022	0.881
CRF	43 (29.5)	21 (25.9)	64 (28.2)	0.32	0.572
AKI (%)	34 (23.3)	38 (46.9)	72 (31.7)	13.427	< 0.001
DM (%)	67 (45.9)	29 (35.8)	96 (42.3)	2.172	0.141
Hypothyroidism (%)	17 (11.6)	5 (6.2)	22 (9.7)	1.782	0.182
Liver injury (%)	69 (47.3)	55 (67.9)	124 (54.6)	8.955	0.003
Site of infection					
RT (%)	103 (70.5)	74 (91.4)	177 (78)	13.136	< 0.001
UT (%)	46 (31.5)	31 (38.3)	77 (33.9)	1.064	0.302
BL (%)	33 (22.6)	29 (35.8)	62 (27.3)	4.572	0.032
GI (%)	34 (23.3)	13 (16.0)	47 (20.7)	1.663	0.197
SST (%)	23 (15.8)	4 (4.9)	27 (11.9)	5.815	0.016
ICU LOS (day)	15 (6, 24)	12 (5, 23.5)	14 (6, 24)	−1.561	0.119
Disease severity					
Sepsis (%)	54 (37.0)	6 (7.4)	60 (26.4)	23.44	< 0.001
Septic shock (%)	92 (63.0)	75 (92.6)	167 (73.6)		
TCM syndrome pattern					
Toxin‐heat syndrome (%)	28 (19.2)	21 (25.9)	49 (21.6)	1.401	0.236
Blood stasis syndrome (%)	16 (11.0)	19 (23.5)	35 (15.4)	6.24	0.012
Bowel qi obstruction (%)	24 (16.4)	12 (14.8)	36 (15.9)	0.103	0.748
Acute deficiency syndrome (%)	78 (53.4)	29 (35.8)	107 (47.1)	6.493	0.011
Laboratory parameters					
CRP (mg/L)	84.3 (39.58, 159.08)	121.3 (47.85, 186.85)	95.1 (40.8, 176.3)	−1.757	0.079
PCT (ng/mL)	1.46 (0.54, 5.68)	3.52 (0.6, 8.36)	2.02 (0.55, 6.64)	−2.386	0.017
WBC (10^9^/L)	10.86 (7.53, 15.36)	13.26 (9.34, 18.38)	11.29 (7.88, 16.27)	−2.47	0.013
NEUT# (10^9^/L)	9.02 (6.15, 13.68)	11.5 (7.71, 15.89)	9.92 (6.32, 14.07)	−2.791	0.005
LYMPH# (10^9^/L)	0.72 (0.4, 1.14)	0.59 (0.34, 1.1)	0.64 (0.36, 1.11)	−1.055	0.291
RBC (10^12^/L)	3.16 (2.61, 3.74)	3.01 (2.28, 3.86)	3.14 (2.46, 3.74)	−0.928	0.353
HGB (g/L)	92 (76.75, 114)	90 (67, 110.5)	91 (74, 113)	−1.291	0.197
PLT (10^9^/L)	32.35 (25.48, 93.5)	43.5 (24.4, 153.5)	33.5 (25.2109)	−0.654	0.513
PT (s)	13.65 (12.1, 15.53)	15.3 (13.05, 18.7)	14.1 (12.5, 16.1)	−3.432	0.001
APTT (s)	31.85 (28.28, 35.68)	35.1 (29.5, 41.05)	33.3 (28.6, 37)	−3.648	< 0.001
TT (s)	14.8 (13.2, 15.93)	16 (14.35, 18.4)	15.2 (13.5, 16.6)	−4.347	< 0.001
FIB (g/L)	3.91 (3.16, 4.91)	3.38 (2.55, 4.42)	3.8 (2.95, 4.69)	−3.267	0.001
FDP (mg/L)	9.59 (5.88, 18.2)	15.5 (7.78, 33)	10.86 (6.36, 22.35)	−3.494	< 0.001
D‐D (mg/L)	1.34 (0.76, 3.04)	2.58 (1.17, 5.22)	1.86 (0.87, 3.59)	−3.558	< 0.001

### 4.2 Univariate Analysis of Factors Influencing Clinical Outcomes in Sepsis Patients

#### 4.2.1 Univariate Logistic Regression Analysis

Based on baseline characteristics, variables demonstrating statistically significant differences between survival and death groups were selected for univariate logistic regression analysis. Additionally, gender, CRP and PLT were included based on clinical relevance. The analysis revealed that gender, age, MAP, PCT, PT, APTT, TT, FIB, WBC, NEUT, disease severity (sepsis vs. septic shock), blood stasis syndrome, RTI, BLI, SSTI, CA, ARDS, pneumonia, AKI and liver injury were all significantly associated with poor prognosis in sepsis patients (all *p* < 0.05) (Table 2).

**TABLE 2:** Univariate logistic regression analysis of risk factors.

Variables	Group	*b*	SE	Wald *χ* ^2^	*p*	OR	95% CI
Gender	Female^a^	−0.776	0.224	11.953	0.001	0.46	0.777–2.383
	Male						
Age		−3.526	0.966	13.334	< 0.001	0.029	1.014–1.063
MAP		−0.028	0.009	9.942	0.002	0.972	0.955–0.989
CRP		0.002	0.001	2.443	0.118	1.002	0.999–1.005
PCT		0.032	0.015	4.822	0.028	1.032	1.003–1.062
PLT		0.001	0.001	0.622	0.43	1.001	0.998–1.004
PT		0.058	0.028	4.112	0.043	1.059	1.002–1.12
APTT		0.057	0.019	9.342	0.002	1.059	1.021–1.098
TT		0.196	0.054	13.225	< 0.001	1.216	1.095–1.352
FIB		−0.307	0.108	8.046	0.005	0.736	0.595–0.91
FDP		0.005	0.004	2.141	0.143	1.005	0.998–1.013
D‐D		0.033	0.022	2.16	0.142	1.033	0.989–1.079
WBC		−1.09	0.268	16.488	< 0.001	0.336	1.004–1.073
NEUT		0.041	0.017	5.785	0.016	1.041	1.008–1.077
Disease severity	No^a^	−2.197	0.43	26.07	< 0.001	0.111	2.992–17.989
	Yes						
Blood stasis syndrome	No^a^	0.912	0.373	5.989	0.014	2.49	1.199–5.17
	Yes						
Acute deficiency syndrome	No^a^	−0.268	0.184	2.121	0.145	0.765	0.278–0.85
	Yes						
RTI	No^a^	−1.815	0.408	19.838	< 0.001	0.163	1.881–10.355
	Yes						
BLI	No^a^	0.647	0.305	4.507	0.034	1.91	1.051–3.47
	Yes						
SSTI	No^a^	−1.281	0.561	5.215	0.022	0.278	0.093–0.834
	Yes						
CA	No^a^	−0.875	0.16	29.756	< 0.001	0.417	2.165–9.174
	Yes						
ARDS	No^a^	1.257	0.376	11.178	0.001	3.516	1.682–7.347
	Yes						
Pneumonia	No^a^	−1.922	0.437	19.331	< 0.001	0.146	1.972–12.083
	Yes						
AKI	No^a^	−0.957	0.179	28.474	< 0.001	0.384	1.628–5.205
	Yes						
Liver injury	No^a^	−1.086	0.227	22.912	< 0.001	0.338	1.337–4.168
	Yes						

*Note:* “a” indicates the control group.

#### 4.2.2 Univariate ROC Analysis

**FIGURE 1:**
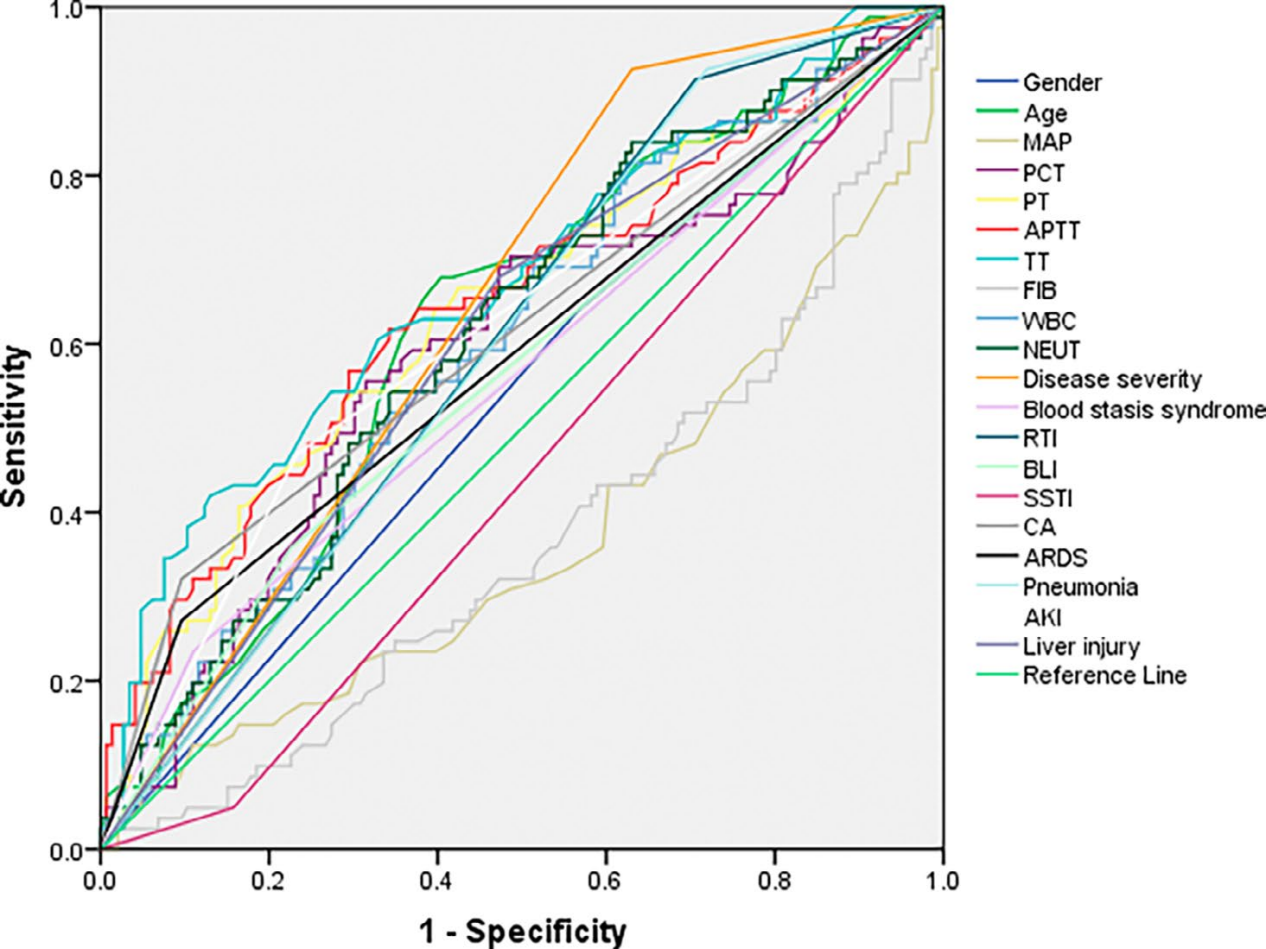
ROC curves of univariate predictors for clinical outcomes in sepsis patients.

Variables showing statistical significance in univariate logistic regression were further analysed using ROC analysis. The results demonstrated that Age, PCT, PT, APTT, TT, WBC, NEUT, Disease severity, RTI, CA, ARDS, pneumonia, AKI and liver injury all exhibited predictive value for clinical outcomes in sepsis patients (AUC > 0.5 and *p* < 0.05 for all) (Figure 1 and Table 3).

**TABLE 3:** Univariate ROC analysis.

Indicators	Cutoff value	Sensitivity	Specificity	Youden index	AUC	95% CI	*p*	SE
Gender	0.5	0.642	0.432	0.074	0.537	0.459–0.615	0.359	0.04
Age	80.5	0.679	0.596	0.275	0.623	0.548–0.698	0.002	0.038
MAP	116.5	0.074	0.952	0.026	0.369	0.291–0.448	0.001	0.04
CRP	102.2	0.605	0.589	0.194	0.57	0.492–0.649	0.079	0.04
PLT	40.75	0.531	0.685	0.216	0.526	0.444–0.608	0.513	0.042
PCT	2.975	0.556	0.685	0.241	0.596	0.516–0.675	0.017	0.04
PT	14.25	0.642	0.603	0.245	0.638	0.56–0.715	0.001	0.04
APTT	34.3	0.617	0.658	0.275	0.646	0.569–0.724	< 0.001	0.04
TT	16.65	0.42	0.87	0.29	0.674	0.6–0.749	< 0.001	0.038
FIB	8.055	0.025	1	0.025	0.369	0.293–0.445	0.001	0.039
WBC	8.955	0.815	0.377	0.192	0.599	0.522–0.676	0.013	0.039
NEUT	6.975	0.84	0.37	0.21	0.612	0.536–0.688	0.005	0.039
Disease severity	0.5	0.926	0.37	0.296	0.648	0.577–0.719	< 0.001	0.036
Blood stasis syndrome	0.5	0.235	0.89	0.125	0.562	0.483–0.642	0.119	0.041
RTI	0.5	0.914	0.295	0.209	0.604	0.53–0.678	0.009	0.038
BLI	0.5	0.358	0.774	0.132	0.566	0.487–0.645	0.1	0.04
SSTI	0.5	0.049	0.842	−0.109	0.446	0.37–0.522	0.177	0.039
CA	0.5	0.321	0.904	0.225	0.613	0.533–0.692	0.005	0.041
ARDS	0.5	0.272	0.728	0.176	0.588	0.508–0.668	0.028	0.041
Pneumonia	0.5	0.926	0.074	0.207	0.603	0.53–0.677	0.01	0.038
AKI	0.5	0.469	0.767	0.236	0.618	0.54–0.696	0.003	0.04
Liver injury	80.5	0.679	0.596	0.275	0.623	0.548–0.698	0.002	0.038

### 4.3 Multivariate Analysis of Factors Influencing Clinical Outcomes in Sepsis Patients

#### 4.3.1 Multivariate Logistic Regression Analysis of Prognostic Factors

Based on the results of the univariate logistic regression analysis, variables with statistically significant differences were selected for multivariate logistic regression analysis. Additionally, based on clinical relevance, CRP and PLT were incorporated into the analysis (Table 4). First, collinearity diagnostics were performed on all included indicators. Blood stasis syndrome, RTI, BLI, pneumonia, AKI and liver injury were ultimately identified as variables causing collinearity and were therefore excluded. The results showed that all variance inflation factor (VIF) values for the remaining 16 indicators were below 5, indicating no significant multicollinearity issues. Consequently, these 16 indicators were ultimately included in the final model.

**TABLE 4:** Collinearity analysis during multivariable logistic regression.

Variables	Unstandardized coefficient		*t*	*p*	Collinearity statistics	
	*B*	SE			Tolerance	VIF
Gender	0.077	0.058	1.328	0.186	0.898	1.113
Age	0.002	0.002	1.102	0.272	0.817	1.224
MAP	−0.002	0.002	−1.04	0.299	0.862	1.16
CRP	0.001	0	2.473	0.014	0.623	1.605
PCT	5.478E‐005	0.003	0.019	0.985	0.814	1.228
PLT	4.147E‐005	0	0.137	0.891	0.836	1.197
WBC	−0.003	0.006	−0.535	0.593	0.234	4.281
NEUT	0.008	0.006	1.479	0.141	0.227	4.406
PT	−0.001	0.004	−0.386	0.7	0.547	1.827
APTT	0.004	0.003	1.078	0.282	0.549	1.82
TT	0.025	0.009	2.792	0.006	0.64	1.562
FIB	−0.051	0.028	−1.85	0.066	0.504	1.983
Disease severity	0.219	0.066	3.342	0.001	0.87	1.15
SSTI	−0.141	0.09	−1.569	0.118	0.853	1.172
CA	0.228	0.074	3.084	0.002	0.915	1.092
ARDS	0.198	0.077	2.562	0.011	0.908	1.101

Multivariate logistic regression analysis revealed that higher CRP levels significantly increased mortality risk (OR = 1.005, 95% CI 1.002–1.009, *p* = 0.003), prolonged TT was associated with increased mortality (OR = 1.172, 95% CI 1.051–1.308, *p* = 0.004), septic shock significantly elevated mortality risk compared to sepsis (OR = 8.265, 95% CI 2.778–24.589, *p* < 0.001), CA was associated with higher mortality (OR = 3.369, 95% CI 1.507–7.529, *p* = 0.003), and ARDS increased mortality risk (OR = 3.377, 95% CI 1.442–7.91, *p* = 0.005) (Table 5). The overall model was statistically significant (Omnibus test of model coefficients, *p* < 0.001) and demonstrated excellent fit (Hosmer‐Lemeshow test, *p* = 0.898). The model's goodness‐of‐fit was further supported by a −2 log‐likelihood value of 220.152, collectively indicating satisfactory model fit.

**TABLE 5:** Multivariate logistic regression analysis of risk factors.

Variables	Group	*b*	SE	Wald *χ* ^2^	*p*	OR	95% CI
CRP		0.005	0.002	8.893	0.003	1.005	1.002–1.009
TT		0.159	0.056	8.149	0.004	1.172	1.051–1.308
FIB		−0.307	0.158	3.784	0.052	0.736	0.54–1.002
Disease severity	No^a^	2.112	0.556	14.416	< 0.001	8.265	2.778–24.589
	Yes						
CA	No^a^	1.215	0.41	8.759	0.003	3.369	1.507–7.529
	Yes						
ARDS	No^a^	1.217	0.434	7.855	0.005	3.377	1.442–7.91
	Yes						

*Note:* “a” indicates the control group.

#### 4.3.2 Multivariate ROC Analysis of Prognostic Factors

**FIGURE 2:**
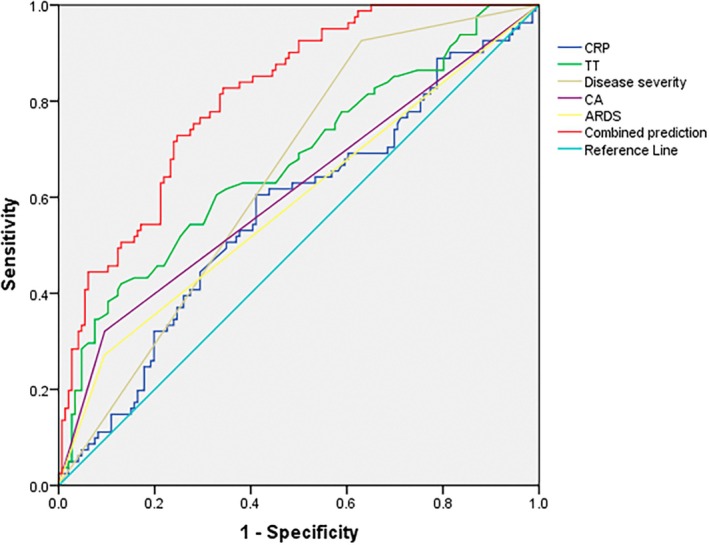
ROC curve analysis of combined prediction for predicting clinical outcomes in sepsis patients.

**FIGURE 3:**
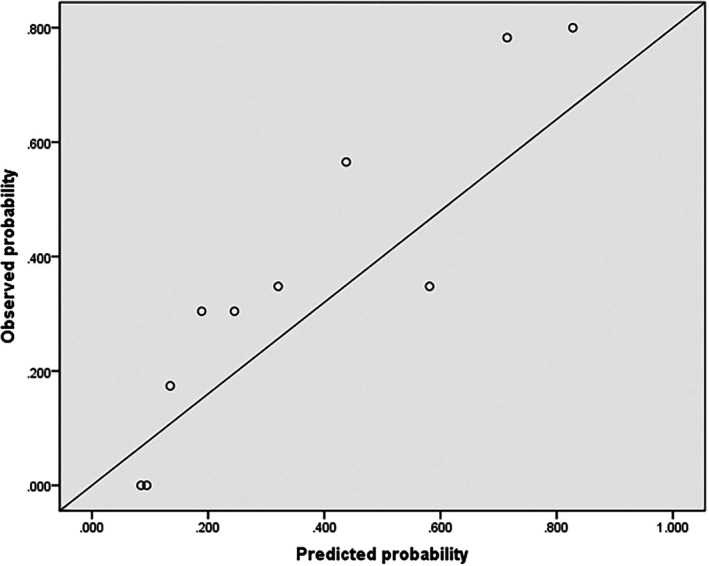
Calibration curve of the combined prediction model.

Based on the aforementioned multivariate logistic regression results, variables demonstrating statistical significance were selected for ROC analysis. The findings revealed that CRP, TT, disease severity, CA and ARDS all possessed predictive value for adverse outcomes in sepsis (all AUC > 0.5). Furthermore, the combined prediction model demonstrated superior performance over any single indicator and achieved moderate predictive value (AUC = 0.812) (Figure 2 and Table 6). The robustness of this combined prediction model was further evaluated through calibration curve analysis. The Hosmer‐Lemeshow goodness‐of‐fit test showed no statistical significance (*p* = 0.067), indicating good model calibration with no significant discrepancy between predicted and observed values (Figure 3).

**TABLE 6:** ROC analysis of combined prediction.

Indicators	Cutoff value	Sensitivity	Specificity	Youden index	AUC	95% CI	*p*	SE
CRP	102.2	0.605	0.589	0.194	0.57	0.492–0.649	0.079	0.04
TT	16.65	0.42	0.87	0.29	0.674	0.6–0.749	< 0.001	0.038
Disease severity	0.5	0.926	0.37	0.296	0.648	0.577–0.719	< 0.001	0.036
CA	0.5	0.321	0.904	0.225	0.613	0.533–0.692	0.005	0.041
ARDS	0.5	0.272	0.904	0.176	0.588	0.508–0.668	0.028	0.041
Combined prediction	—	0.827	0.658	0.485	0.812	0.757–0.867	< 0.001	0.028

## 5 Discussion

This study enrolled a total of 227 sepsis patients, with a higher proportion of males (59.5%), which is consistent with previously reported gender epidemiological characteristics in sepsis studies [13, 14, 15]. However, there remains no consensus regarding how gender influences sepsis prognosis. Some studies suggest that male sepsis patients have higher mortality rates compared to females [16]. Two potential explanations have been proposed for this phenomenon: first, higher oestrogen levels in females may enhance immune function; second, the predominance of anti‐inflammatory mediators in females might provide protective effects in critically ill patients [17, 18, 19]. In contrast, another study found no significant gender difference in overall sepsis mortality (male 20.1% vs. female 19.8%, *p* = 0.834), but observed significantly higher mortality in females with severe sepsis (including septic shock) (female 63.5% vs. male 46.4%, *p* = 0.007) [13]. In our study, univariate logistic regression analysis initially identified female gender as significantly associated with poor prognosis (*p* = 0.001), and univariate ROC analysis showed age had modest predictive value for outcomes (AUC = 0.623 > 0.5). However, multivariate logistic regression revealed no significant association between gender and prognosis, which may be attributed to the limited sample size in the death group of our study.

Association between comorbidities and clinical outcomes. Our univariate and multivariate logistic regression analyses demonstrated that sepsis patients with concomitant CA or ARDS had significantly worse clinical outcomes. Existing literature indicates that many elderly sepsis patients have underlying coronary artery disease [20]. The hemodynamic alterations induced by sepsis—including vasodilation and hypovolaemia—can further compromise myocardial perfusion in these vulnerable patients, ultimately precipitating CA [21]. Post‐CA, patients develop a pathological state termed ‘post‐cardiac arrest syndrome,’ characterised by brain injury, myocardial dysfunction, systemic ischemia–reperfusion injury and persistent precipitating conditions. This pathophysiology has been described as a ‘sepsis‐like’ syndrome [22, 23], which may be more pronounced in the setting of persistent septic shock [21]. Additionally, infections are recognised as a major complication following CA, with post‐arrest sepsis and septic shock associated with significantly elevated mortality [24, 25]. ARDS, a severe acute inflammatory lung injury, affects approximately 30% of sepsis patients in China and exacerbates disease severity, contributing to poor outcomes [26, 27, 28, 29]. Previous studies report that sepsis patients with ARDS have an in‐hospital mortality rate of 60%—four times higher than those without ARDS (14%)—with mortality risks ranging from 20% to 50% [30, 31].

Septic shock is a critical condition that develops from sepsis and is characterised by severe circulatory dysfunction and cellular metabolic disturbances, often accompanied by coagulation abnormalities [1, 32]. Reported mortality rates for sepsis range from 25% to 30%, while mortality for septic shock is significantly higher, reaching 40% to 50% [33]. In this study, multivariable logistic regression analysis confirmed that septic shock is an independent risk factor for adverse outcomes in septic patients, consistent with previous findings [34, 35].

Regarding the relationship between TCM syndrome types and prognosis, previous studies have suggested that blood stasis pathology is involved in the process of sepsis and is associated with coagulation dysfunction [33, 36, 37], with some clinical studies indicating its prognostic value [34, 35]. A preliminary theoretical framework has been established regarding the association between blood stasis syndrome and coagulation dysfunction. Studies consistently observe that patients with blood stasis syndrome present with typical coagulopathy manifestations, including decreased PLT, prolonged PT, APTT and TT, as well as elevated D‐D levels [38, 39]. Therapeutically, Chinese herbal medicines with blood‐activating and stasis‐resolving properties have been demonstrated to exert multiple effects, including improving coagulation parameters, regulating microcirculation, increasing tissue perfusion, alleviating vasospasm and inhibiting microthrombus formation [40, 41]. However, current research primarily focuses on phenomenological observations and clinical efficacy validation. The specific molecular mechanisms and signalling pathway networks through which blood stasis syndrome influences coagulation function have not been systematically investigated, and its deeper biological basis warrants further exploration.

In our attempt to incorporate ‘blood stasis syndrome’ in the multivariable model, we identified complex statistical collinearity between this TCM syndrome and a series of indicators reflecting systemic infection and organ dysfunction. As a result, it was not retained as an independent predictor in the final model. Notably, univariate analysis in our study revealed an association between blood stasis syndrome and adverse outcomes (OR = 2.49, 95% CI 1.199–5.17, *p* = 0.014), and ROC curve analysis indicated some predictive capacity (AUC > 0.5). These results suggest that although blood stasis syndrome did not demonstrate independent predictive value in the multivariable analysis, its prognostic role at the univariate level remains noteworthy. The findings of this study provide new perspectives on the role of TCM syndromes in sepsis prognosis assessment. Through systematic statistical analysis, we observed complex correlative characteristics between blood stasis syndrome and modern critical illness indicators. Specifically, the predictive value of blood stasis syndrome in univariate analysis and its network collinearity with critical indicators reflect, at a data level, an inherent consistency between TCM macro‐pattern differentiation and Western medicine micro‐indicators in evaluating sepsis prognosis. This discovery not only deepens the understanding of TCM pathogenesis characteristics in sepsis but also provides methodological references for constructing an integrated prognostic assessment framework that combines the strengths of both TCM and Western medicine, representing an innovative advancement compared to conventional biomarker studies.

Association of laboratory parameters with clinical outcomes. Among various laboratory indicators, CRP has been widely utilised in the diagnosis, treatment and prognostic evaluation of sepsis [42]. Multiple studies have demonstrated a significant correlation between elevated CRP levels and poor clinical outcomes in sepsis patients [43, 44, 45]. Our multivariate logistic regression analysis further substantiated this finding, identifying CRP as an independent risk factor for sepsis‐related mortality with modest predictive value (AUC = 0.57 > 0.5). During sepsis progression, systemic inflammatory responses can induce vascular endothelial injury and platelet activation, ultimately leading to coagulation dysfunction [46]. These hematologic disturbances typically manifest as prolonged PT, APTT and TT, along with elevated FDP and decreased FIB levels [47, 48]. Notably, FIB—a major indicator of sepsis‐induced coagulopathy—functions as an acute‐phase protein whose levels may rise compensatorily during inflammation [49]. Consequently, FIB concentrations may remain within normal ranges despite ongoing consumption, necessitating serial monitoring for accurate clinical assessment [50]. Our univariate analysis identified both TT and FIB as independent risk factors for sepsis mortality.

Finally, we constructed a ROC curve for the combined prediction model incorporating CRP, TT, disease severity, CA and ARDS. The results demonstrated that the combined model exhibited moderate predictive performance compared to any individual indicator, with acceptable model fit.

## 6 Conclusions

This retrospective study established conventional indicators (CRP, TT, sepsis severity, CA, ARDS) as independent mortality risk factors in sepsis, with combined prediction showing moderate performance. Furthermore, this study demonstrates an inherent consistency between TCM macro‐pattern differentiation and Western medical indicators in sepsis prognosis assessment, as evidenced by the predictive value of blood stasis syndrome in univariate analysis and its network collinearity with critical indicators. These findings provide a methodological framework for developing integrated prognostic models that combine the strengths of both medical systems, representing an innovative advancement beyond conventional biomarker studies.

However, this study has several limitations: First, as a single‐centre retrospective analysis with a relatively small sample size, the findings may lack generalizability. Second, some medical records were incomplete or inconsistently documented. Third, although multivariate regression was employed to control for confounding factors, residual bias from unmeasured or unknown variables may persist. Future research should prioritise prospective, multicentre, large‐scale randomised controlled trials with standardised data collection protocols. Advanced statistical approaches, such as instrumental variable analysis and incorporation of additional key variables, should be implemented to better control for potential biases and validate the reliability of these findings.

## Author Contributions

**Mengxia Yang:** writing – original draft, methodology, formal analysis. **Tengfei Chen:** methodology, formal analysis. **Junhao Liu:** visualization, methodology. **Xiaolin Wang:** visualization. **Xuerui Wang:** review and editing. **Xiaolong Xu:** supervision. **Qingquan Liu:** funding acquisition, supervision.

## Funding

The present study was supported by the National Natural Science Foundation of China (NSFC) General Program (grant no. 82474428), the National Natural Science Foundation of China (NSFC) General Program (grant no. 82575203), the National Major Science and Technology Project (2025ZD01903000), and the National Major Science and Technology Project (2025ZD01903100).

## Ethics Statement

This study was approved by the Ethics Committee of Beijing Hospital of Traditional Chinese Medicine affiliated with Capital Medical University (Approval No. 2024BL02‐099‐01).

## Conflicts of Interest

The authors declare no conflicts of interest.

## Data Availability

The data that support the findings of this study are available from the corresponding author upon reasonable request.
